# Disseminated peritoneal hydatidosis following blunt abdominal trauma: A case report

**DOI:** 10.1186/1757-1626-1-118

**Published:** 2008-08-21

**Authors:** Nimish J Shah, Nikunj K Vithalani, Rahul K Chaudhary, Prashant N Mohite

**Affiliations:** 1 Associate Professor, Department of Surgery, Government Medical College & Sir Sayajirao General Hospital, Baroda, Gujarat, India; 2 Resident, Department of Surgery, Government Medical College & Sir Sayajirao General Hospital, Baroda, Gujarat, India; 3 Current affiliation: Senior Resident, Department of Surgical Oncology, Morbai Naraindas Cancer Institute, Inlaks and Budhrani Hospital, Pune, Maharashtra, India; 4 Current affiliation: Senior Resident, Department of Cardiothoracic and Vascular Surgery, Postgraduation Institute of Medical Education & Research, Chandigarh, India

## Abstract

A middle age lady presented with abdominal pain was diagnosed to have multiple peritoneal and hepatic hydatid cysts on CT scan. Retrospectively she was found to have suffered blunt abdominal trauma.

## Case presentation

**Figure 1:**
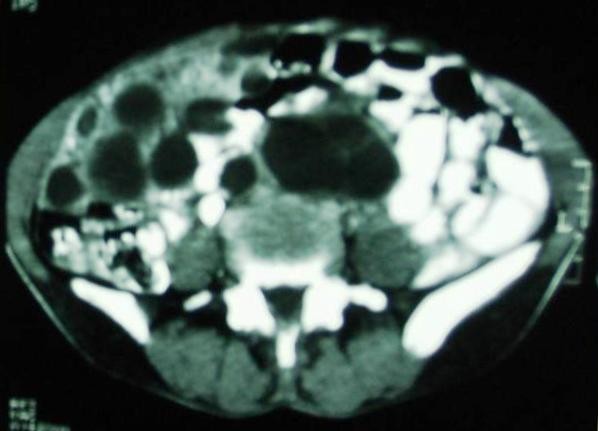
CT scan showing multiple thin walled cysts.

A forty five year old lady presented with chronic dull aching pain with gradually increasing lump in the epigastric and pelvic region since 3 years. A well defined, firm mass of about 20 × 20 centimeters was palpable in epigastrium extending more on right hypochondrium, probably arising from left lobe of liver. A similar mass was palpable in the hypogastrium while multiple small nodular masses palpable in the right iliac fossa and lumbar region. Detailed inquest revealed a 5 year old incidence of blunt abdominal trauma, while working in her farm. The ultrasonography and CT scan (See Figure 1) showed multiple thin walled cysts of varying size involving left lobe of liver, peritoneal cavity, omentum and mesentery. Cysts showing internal septae and peripheral tiny calcific foci were also seen extending into pelvis around uterus, adnexae and retro-uterine cervical region, markedly compressing distal descending colon, sigmoid colon and rectum. Ileal loops were compressed and displaced superiorly. Serology for hydatid cyst disease was positive with ELISA test.

**Figure 2:**
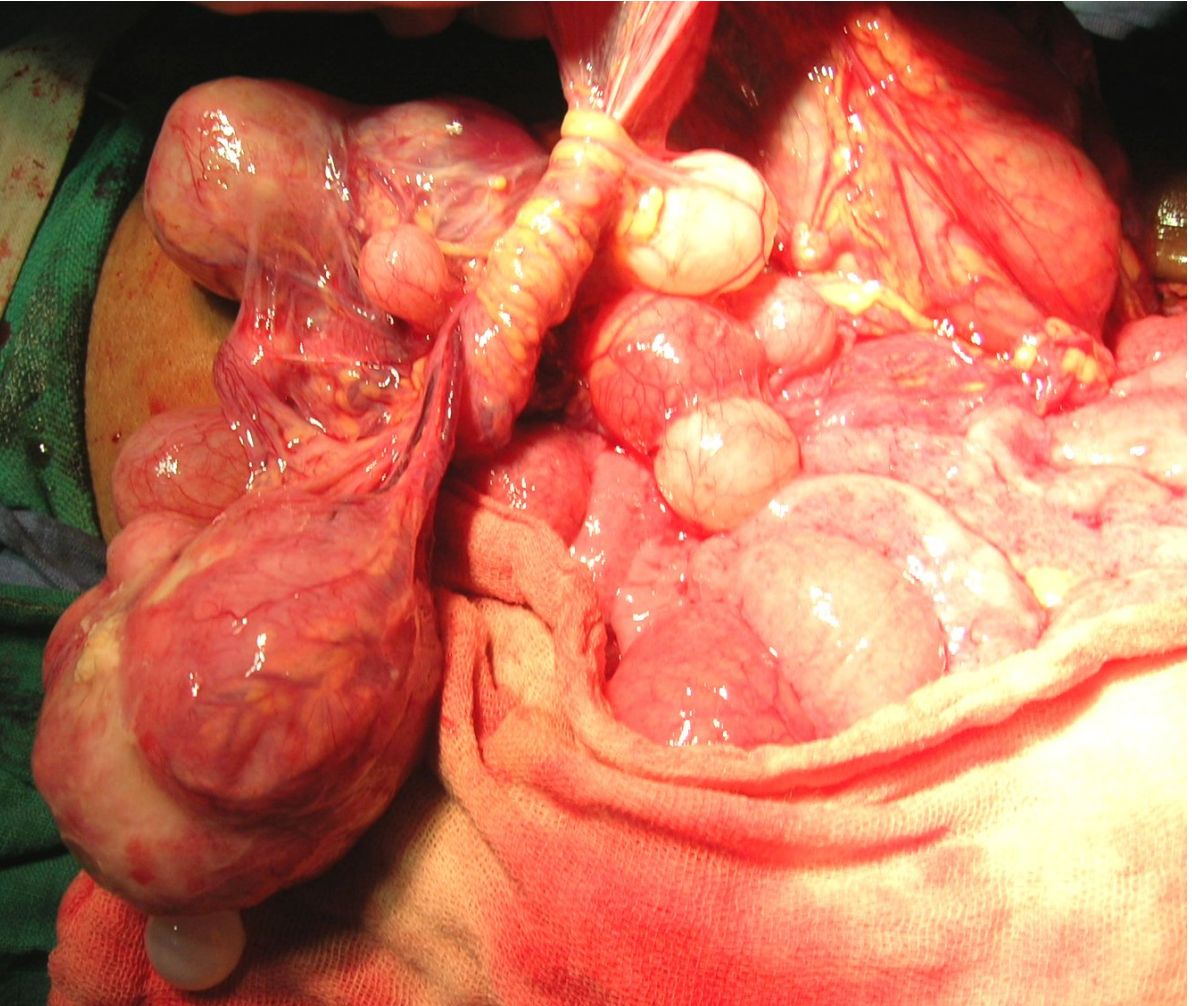
Peritoneal cavity crammed with cysts.

**Figure 3:**
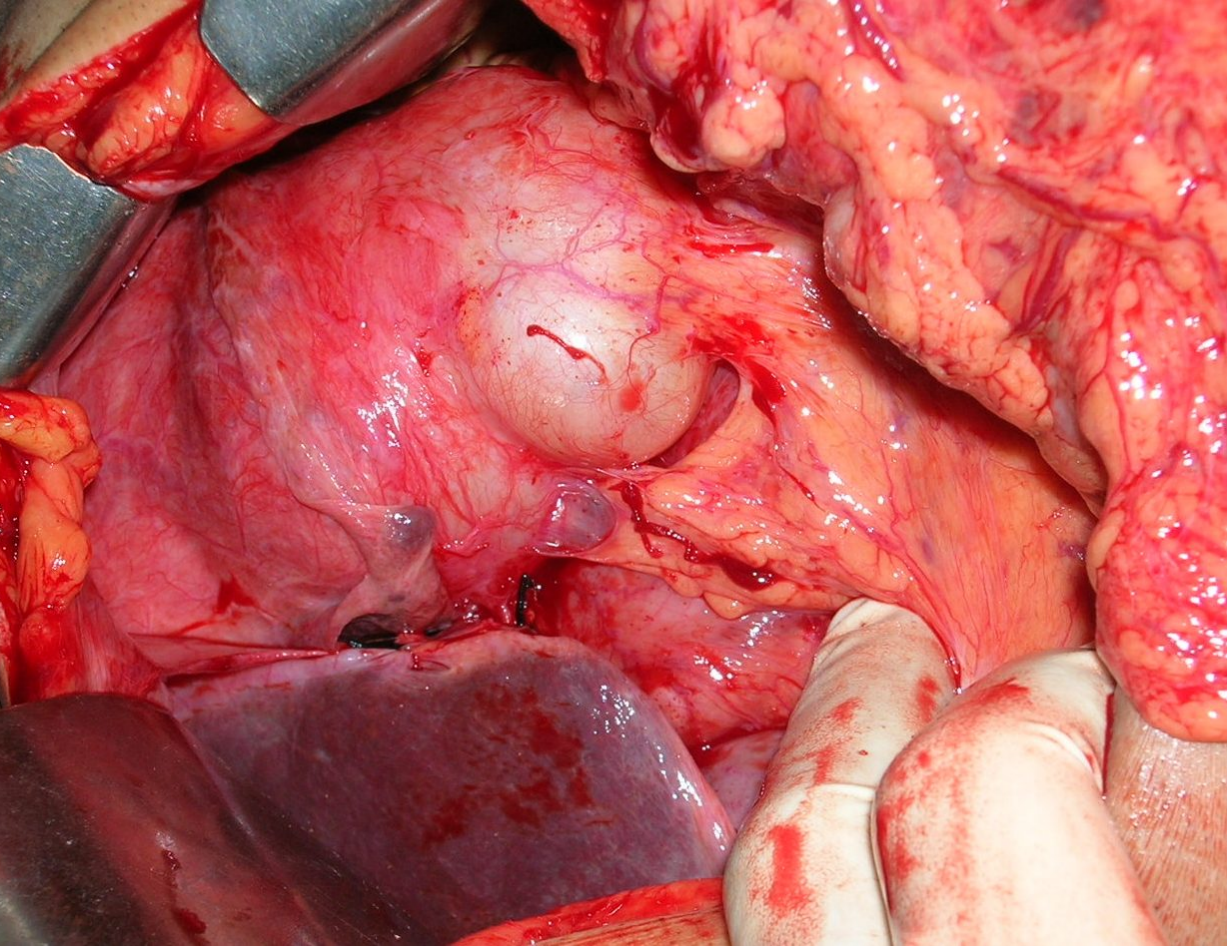
Omentum was studded with cysts.

**Figure 4:**
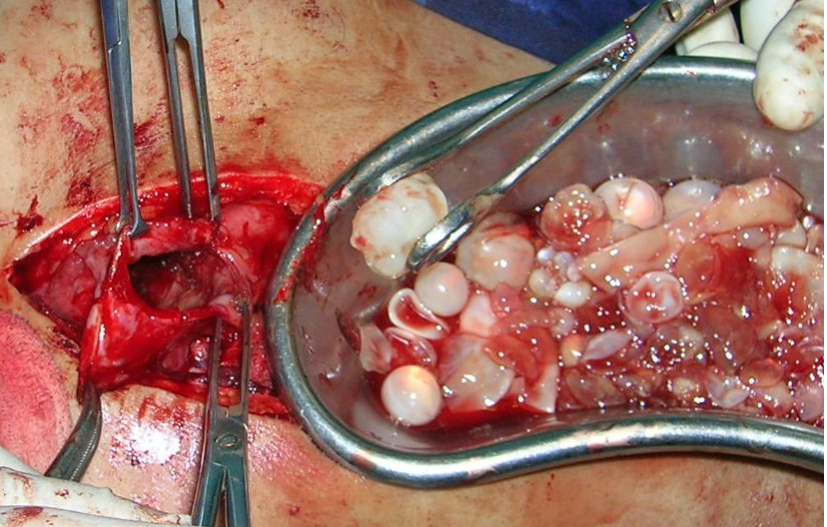
Berry picking of cysts.

**Figure 5:**
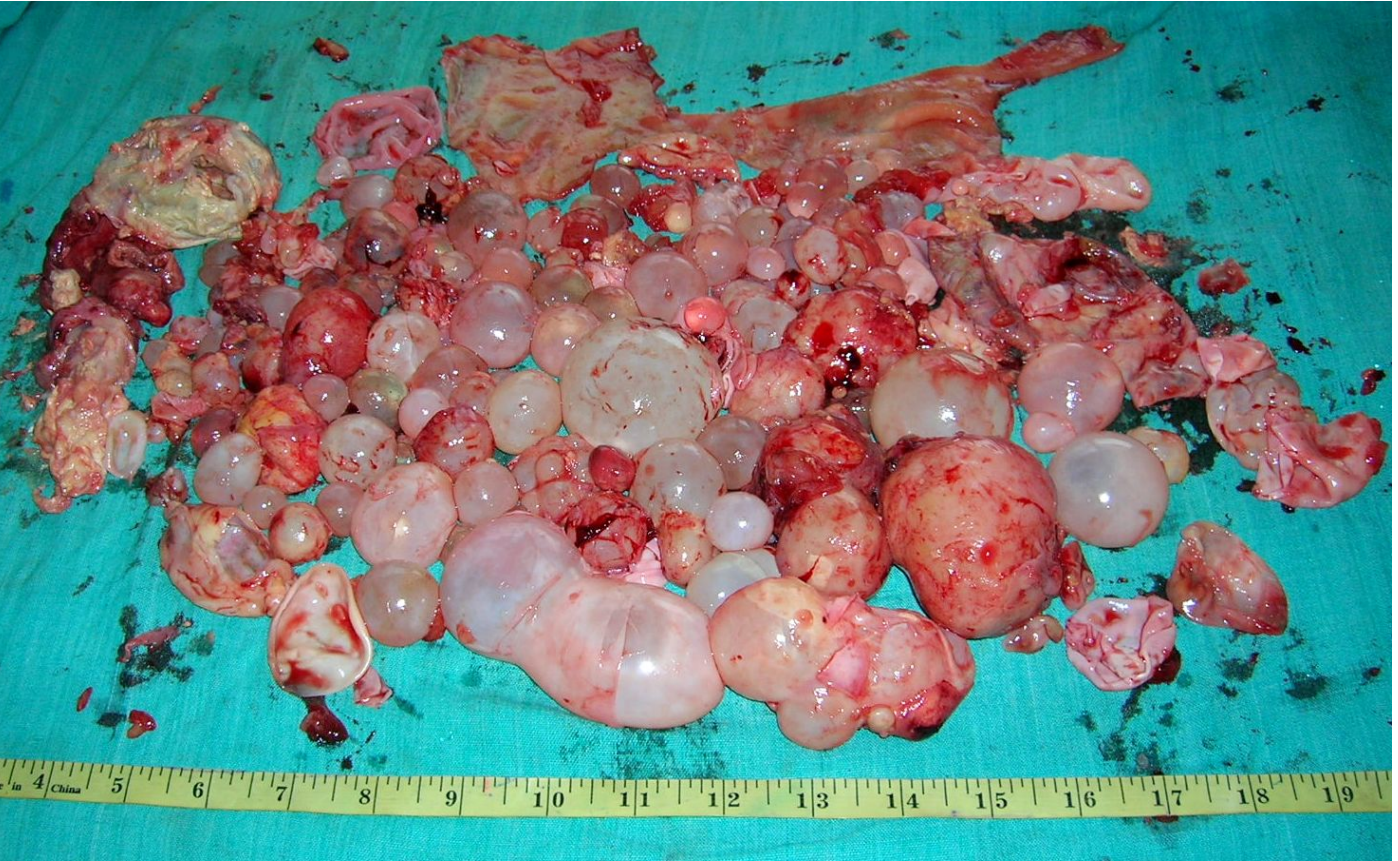
Cysts removed from peritoneum.

Two courses of 4 weeks of Albendazole (15 mg/kg/day) were given with the interval of 1 month. The follow up after 1 month did not show any decrease in the size of the cysts and decision of laparotomy was taken. Peritoneal cavity was crammed with cysts ranging from 1 to 12 centimeters (See Figure 2). Omentum was studded with cysts (See Figure 3) which was incised and cysts were picked up (See Figure 4). A superficial cyst found in the left lobe of liver was opened with small incision and the hydatid fluid and daughter cysts were drained. After the excision of germinal membrane the cavity was masupialized. Later, the pelvic cavity was exposed and cysts adherent to adnexa, uterus, broad ligament, urinary bladder and rectum were removed. More than 250 cysts of different sizes were removed from the abdomen (See Figure 5). Patient died of anaphylactic shock within few hours of operation.

## Discussion

Hydatid cyst disease is a zoonotic disease caused by the larval stage of Echinococcus granulosus (dog tapeworm), E. multilocularis, or E. vogeli [1]. This disease occurs when humans ingest the hexacanth embryos of the dog tape worm. Infestation by hydatid disease in humans most commonly occurs in the liver (55–70%) followed by the lung (18–35%); the two organs can be affected simultaneously in about 5–13% of cases [2].

Peritoneal hydatidosis is almost always secondary to hepatic disease, although some unusual cases of primary peritoneal hydatidosis have been described. The overall frequency of peritoneal disease in cases of abdominal echinococcus is approximately 13%. Peritoneal involvement is usually undetected unless cysts are large enough to cause symptoms. Most of the cases of peritoneal hydatid disease are secondary to previous surgery for liver hydatidosis. In present case blunt abdominal injury was the probable cause of dissemination. Systemic anaphylaxis is usually associated with cyst rupture and can be predicted by positivity of Casoni reaction.

USG is the first line of screening for abdominal hydatidosis. USG is particularly useful for detection of cystic membrane, septa, and to look for hydatid sand. CT scan best demonstrates cyst wall calcification and cyst infection [3]. Immunoelectrophoresis, enzyme-linked immunosorbent assay (ELISA), latex agglutination and indirect haemagglutination (IHA) test are being carried out for the diagnosis, screening and post-operative follow up for recurrence [4].

The treatment of choice for localized hydatid cysts in liver or lungs is principally surgical while the therapy for disseminated peritoneal hydatidosis remains medical [5]. Therapy with albendazole or praziquantel remains the mainstay of medical therapy. After medical treatment, the hydatid cysts show gradual reduction in cyst size and number and the follow up is advisable with Ultrasonography or CT scan. In our case despite of sufficient medical treatment the cyst size and number did not reduced and hence surgery remained the final resort. Surgery can be performed with removal of the cyst after sterilizing the cyst with formalin or alcohol. However, pre- and post-operative 1-month courses of albendazole or 2 weeks of praziquantel should be considered in order to sterilize the cyst, decrease the chance of anaphylaxis, decrease the tension in the cyst wall and to reduce the recurrence rate post-operatively [6]. Intra-operatively, the use of hypertonic saline or 0.5% silver nitrate solutions before opening the cavities tends to kill the daughter cysts and therefore prevent further spread or anaphylactic reaction.

Anaphylctic shock occurs when there is prior sensitization. The present case might have been sensitized at the time of rupture of hepatic cyst when she sustained blunt abdominal trauma. Utmost care of preventing the contact of hydatid cyst contents with the body tissues during operation can not avoid the anaphylactic shock. The surgeon should be ready for this catastrophe while operating hydatid cyst especially with the suspicion of prior sensitization.

## Consent

A written consent was taken from the patient and her father regarding the publishing of this article.

## Competing interests

The authors declare that they have no competing interests.

## Authors' contributions

All the authors read and approved the final manuscript. NJS did the surgery, RKS and NV assisted the surgery. PNM is the corresponding author of the article who drafted and finalized the manuscript.
